# From cavities to canals: evidence of developmental continuity in *Myrsine guianensis* (Primulaceae)

**DOI:** 10.1007/s10265-026-01732-1

**Published:** 2026-07-20

**Authors:** Silvia Rodrigues Machado, Zoraide Valério, Karla Bianca de Deus Bento, Yve Canaveze

**Affiliations:** 1 Institute of Biosciences of Botucatu (IBB), Department of Biodiversity and Biostatistics, São Paulo State University (UNESP), Botucatu, São Paulo Brazil; 2 Graduate Program in Plant Biology, Botucatu/Rio Claro Interunit, São Paulo State University (UNESP), Botucatu, São Paulo Brazil; 3 Department of Botany, Federal University of São Carlos (UFSCar), São Carlos, São Paulo Brazil

**Keywords:** Cell turnover, Cell wall remodeling, Oil secretion, Resin secretion, Secretory spaces, Transitional forms

## Abstract

Secretory cavities and canals are traditionally treated as distinct anatomical categories and widely employed as diagnostic characters in taxonomic studies. However, their coexistence or the presence of intermediate forms in the same individual or organ points to a more intricate developmental relationship. The mechanisms underlying their formation and structural differentiation, particularly the boundaries between cavities and canals, remain insufficiently understood. In this study, we selected *Myrsine guianensis* (Aubl.) Kuntze (Primulaceae) because it displays globose and elliptical cavities alongside linear, canal-like secretory spaces. This condition raises a fundamental question: do these structures represent two discrete types of secretory spaces, or are they transitional forms along a developmental continuum? To address this question, we investigated the secretory spaces from a developmental point of view using light and transmission electron microscopy. Secretory cavities originated from the fundamental meristem through a schizolysigenous process, giving rise to a lumen lined by a uniseriate secretory epithelium and surrounded by one or two layers of sheath cells. Epithelial cells exhibited ultrastructural features indicative of intense metabolic activity associated with oil-resin synthesis. Secretion involved pronounced cell-wall remodeling, transient periplasmic spaces, and the replacement of senescent epithelial cells. A shift from resin- and phenolic-rich secretion to predominantly oil-rich secretion coincided with epithelial senescence and the recruitment of sheath cells, thereby sustaining secretory activity. The fusion of adjacent cavities, epithelial reorganization, and progressive lumen expansion produced transitional forms between globose and elongated structures, supporting the existence of a developmental cavity-canal continuum. This structural plasticity challenges the view of secretory spaces as discrete anatomical entities and suggests that spatial constraints during morphogenesis contribute to their diversification.

## Introduction

Plant secretory structures exhibit remarkable morphological and anatomical diversity and are widely employed as taxonomic characters due to their consistent position within species ( Fahn 1979, 1988; Metcalfe and Chalk 1950). Among internal secretory systems, canals and cavities are glands formed by intercellular spaces lined with specialized secretory cells (secretory epithelium) and commonly surrounded by a sheath of non-secretory cells (Evert 2006).

Distinguishing between these two types of structures remain challenging, mainly because of their structural similarity, especially the presence of a lumen lined by a secretory epithelium (Martinez-Quesada et al. 2025). Although several criteria have been proposed to differentiate them (e.g., Martínez-Quezada et al. 2025; Paiva et al. 2026; Prado and Demarco 2018; Turner et al. 1998), the most reliable approach is the combined analysis of transverse and longitudinal sections, which enables a more accurate understanding of their organization. Secretory cavities are isodiametric, with a spherical to elliptical shape, whereas secretory canals are elongated, tubular structures that may extend throughout the plant body (Evert 2006). This distinction, however, is not always straightforward. In some cases, secretory spaces originate as discrete cavities separated by epithelial cells and later undergo elongation through the stretching and separation of intervening septa (Lersten and Curtis 1989). As a result, mature structures may give the misleading impression of a single, continuous canal, when they represent a series of interconnected tubular cavities (Lersten and Curtis 1989).

Canals are broadly distributed among angiosperms, whereas cavities are more restricted, occurring in families such as Apocynaceae, Asclepiadaceae, Asteraceae, Fabaceae, Malvaceae, Myrtaceae, Primulaceae, Rutaceae (Evert 2006; Metcalfe and Chalk 1950).

Within Primulaceae, the presence of secretory cavities has diagnostic value for distinguishing two subclades: Primuloideae, comprising herbaceous species lacking secretory cavities, and Myrsinoideae, which includes genera characterized by their presence (Judd et al. 2009). In Myrsinoideae, these cavities are of schizogenous origin and secrete resinous material with a yellowish to orange coloration, predominantly composed of hydroxybenzoquinone derivatives, whose composition varies among species (Metcalfe and Chalk 1950; Otegui et al. 1998a, b).

The genus *Myrsine* L. comprises approximately 300 species, of which 26 occur in Brazil (Freitas 2012). Popularly known as “capororoca,” these plants are used in folk medicine to treat diseases affecting both animals and humans (Bueno et al. 2005). Species of this genus are notable for their chemical diversity, with more than 130 identified compounds, including essential oils, flavonoids, saponins, and quinones, many of which exhibit antioxidant, anti-inflammatory, and antimicrobial activities (Costa et al. 2020; França et al. 2011; Thoa and Son 2024). The leaves of *Myrsine* species display translucent, orange to blackish punctations when observed against the light (Freitas and Carrijo 2008), corresponding to secretory cavities and duct-like structures located in the mesophyll and petiole (De Luna et al. 2014; Luna et al. 2019).

Despite the ecological, evolutionary, and chemotaxonomic significance of secretory cavities in Myrsinoideae (Ogawa and Natori 1968; Otegui et al. 1998b), most studies have focused primarily on their formation and early developmental stages. Integrative approaches that encompass ontogenetic, structural, and functional perspectives, tracking secretory cavities from their origin to full maturity and activity, remain scarce, highlighting a critical need for comprehensive developmental investigations.

In this context, we investigated *Myrsine guianensis* (Aubl.) Kuntze, a shrub or small tree widely distributed in forest and savanna vegetation types in Brazil (Freitas 2012), which exhibits both globose to elliptical cavities and elongated, canal-like secretory spaces containing dark secretion (Freitas 2012; Freitas 2026; Freitas and Kinoshita 2015; Otegui 1998; Otegui and Maldonado 1998). This condition raises a central question: do these structures represent distinct categories or transitional forms within a developmental continuum? To address this issue, we analyzed the ontogeny and ultrastructure of the secretory spaces of this species using light and transmission electron microscopy.

## Materials and methods

### Plant material and collection site

Samples were collected from at least three individuals of *M. guianensis* growing in a fragment of cerrado *sensu stricto* located in Botucatu municipality (22°53′25′′ S and 48°27′19′′ W), southeastern Brazil, between November 2023 and February 2025. This vegetation type represents the typical savanna physiognomy of the Brazilian Cerrado, characterized by low, sparse trees with twisted trunks and thick bark, occurring on well-drained soils, with woody cover ranging from 5 to 70% and a discontinuous shrub-herbaceous layer. Cerrado soil is sandy, dystrophic, acidic, has high levels of aluminum, and the vegetation is adapted to xeric conditions (Gottsberger and Silberbauer-Gottsberger 2006). The average annual temperature is 21.34 °C, and the average annual precipitation is 1,500 mm in the region, with a well-defined dry season from May to September. According to the Köppen climate classification, the region’s climate has recently been classified as Aw, with hot, humid summers and cold, dry winters (Franco et al. 2023).

In the study area, *M. guianensis* occurred as evergreen shrubs 1.5–2.0 m tall. A voucher (36215) was deposited in the BOTU Herbarium of the Institute of Biosciences of Botucatu (IBB), São Paulo State University (UNESP).

For microscopic analyses, samples of dormant apical buds (including the shoot apex, leaf primordia, and undifferentiated leaves) were collected, in addition to unexpanded leaves (2–4 cm long) and fully expanded leaves (8–9 cm long) produced at the beginning of the budding cycle. For each developmental stage, three samples were collected from five individuals. Immediately after collection, the material was dissected, and fragments were fixed in appropriate media for anatomical, histochemical, and ultrastructural analyses.

### Structural, histochemical and ultrastructural studies

For light microscopy (LM) analyses, samples were fixed in Karnovsky’s solution (Karnovsky 1965) for 24 h, dehydrated through a graded ethanol series, and embedded in (2-hydroxyethyl) methacrylate historesin (Leica Microsystems). Cross and longitudinal Sects.  (4–6 μm thick) were obtained using a semi-automatic rotary microtome (Leica RM2245), stained with 1% toluidine blue in 1% aqueous solution (O’Brien et al. 1964), and mounted in synthetic resin (Entellan, Merck).

Histochemical tests were performed on fresh material sectioned at 10–12 μm using a hand microtome. The following reagents were applied: Sudan IV for total lipids (Johansen 1940); Nadi reagent (α-naphthol and p-dimethylphenylenediamine) for essential oils and oleoresins (David and Carde 1964); ferric chloride for phenolic compounds (Johansen 1940); ruthenium red for pectic substances (Jensen 1962); and tannic acid followed by ferric chloride for mucilage (Pizzolato and Lillie 1973). Control sections were prepared according to the specifications of each histochemical assay. Observations and documentation were performed using a light microscope (Leica DMR) equipped with a digital camera (Leica DFC 500).

For transmission electron microscopy (TEM), samples were fixed overnight at 4 °C in 2% (v/v) glutaraldehyde and 4% (w/v) paraformaldehyde prepared in 50 mM sodium phosphate buffer (pH 7.4). The material was post-fixed in 1% (w/v) osmium tetroxide in the same buffer for 2 h at 24 °C, dehydrated in a graded acetone series, and embedded in Araldite resin (Araldite 502, Electron Microscopy Sciences). Ultrathin sections (60–70 nm thick) were obtained using an ultramicrotome (Leica Reichert Ultracut S), collected on copper grids, and contrasted with 4% (w/v) uranyl acetate (Watson 1958) and lead citrate for 15 min (Reynolds 1963). Sections were examined using a transmission electron microscope (Tecnai Spirit, FEI Company) operated at 80 kV.

## Results

**Fig. 1:**
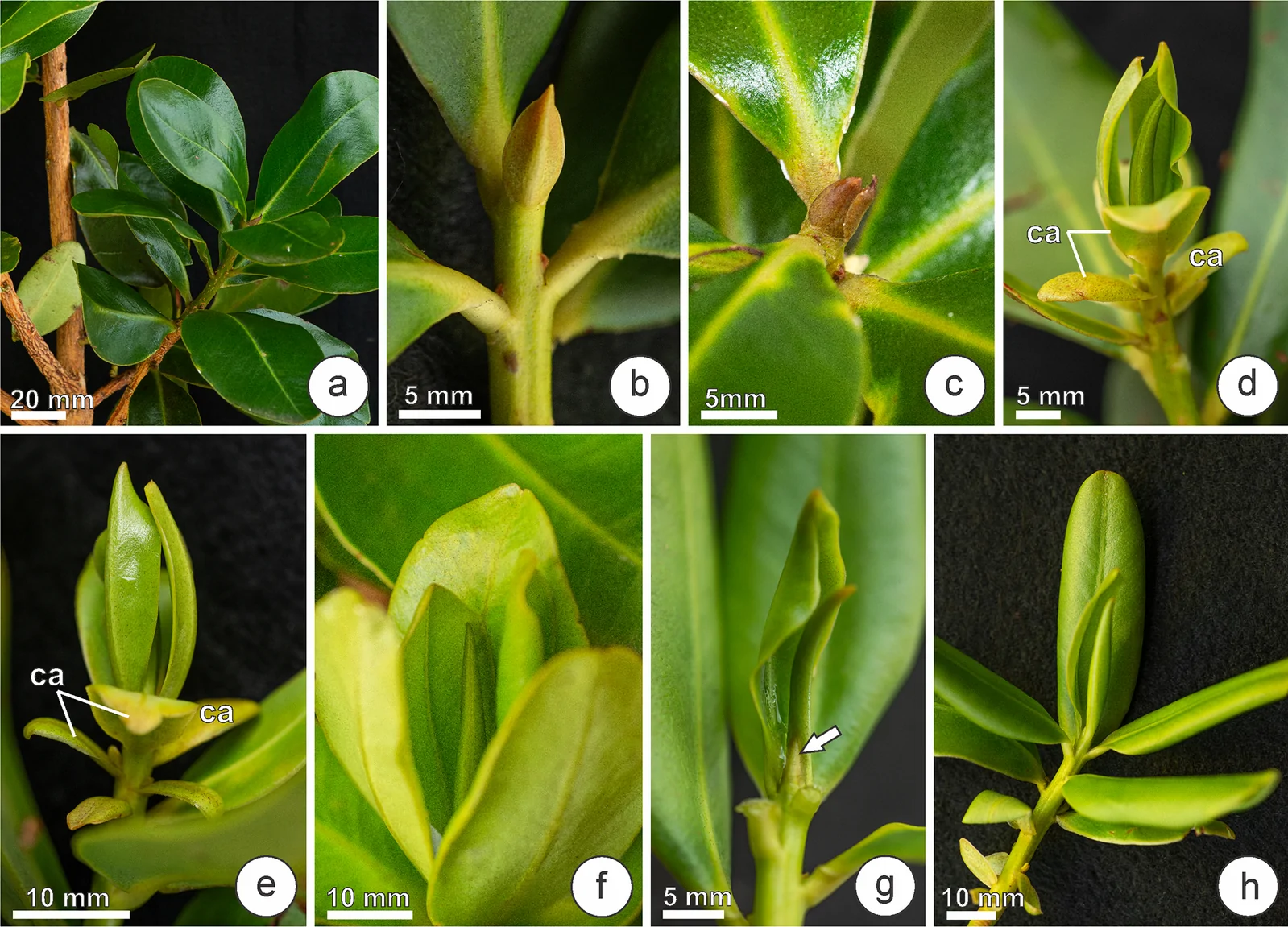
Shoot apex and leaf morphology of *Myrsine guianensis*. (**a**) Vegetative stem from the previous growing season; brown fissured bark; dark green, chartaceous, glossy leaves. (**b**) Dormant shoot apex enclosed by cataphylls. (**c**) Shoot apex at the onset of growing season; cataphylls separating. (**d**) Cataphylls at the base and surrounding young leaves. (**e**, **f**) Newly formed leaves erect, conduplicate, partially overlapping vegetative apex. (**g**) Leaves removed to expose the shoot apex (arrow) encircled by conduplicate leaves. (**h**) Young branch from the current growing season; leaves membranous, margins distinctly conduplicate. *Symbols*: ca. = cataphyll

Individuals of the *M*. *guianensis* retained leaves year-round, with regrowth events that can extend over several months, especially in spring and summer. The terminal branches exhibited thick bark and leathery, bright green leaves with alternating phyllotaxis. The dormant apical bud was protected by cataphylls, which were yellowish to brownish and bear numerous brown spots (Fig. 1b). When the vegetative apex resumed activity, the cataphylls separated (Fig. 1c) but persisted on the branch. The cataphylls remaining have reduced size, greenish-yellow color, blackened spots, and coriaceous texture (Fig. 1d, e). Newly formed leaves were erect, conduplicate, and partially overlapped the vegetative apex (Fig. 1e–g). The vegetative branches produced in the previous growing season exhibited short internodes, fissured brown bark and dark green leaves with a glossy sheen (Fig. 1a), while those formed during the current growing season exhibit elongated internodes and a smooth surface, bearing leaves with a membranous texture and distinctly conduplicate margins (Fig. 1h).

### Development, structure and histochemistry

**Fig. 2:**
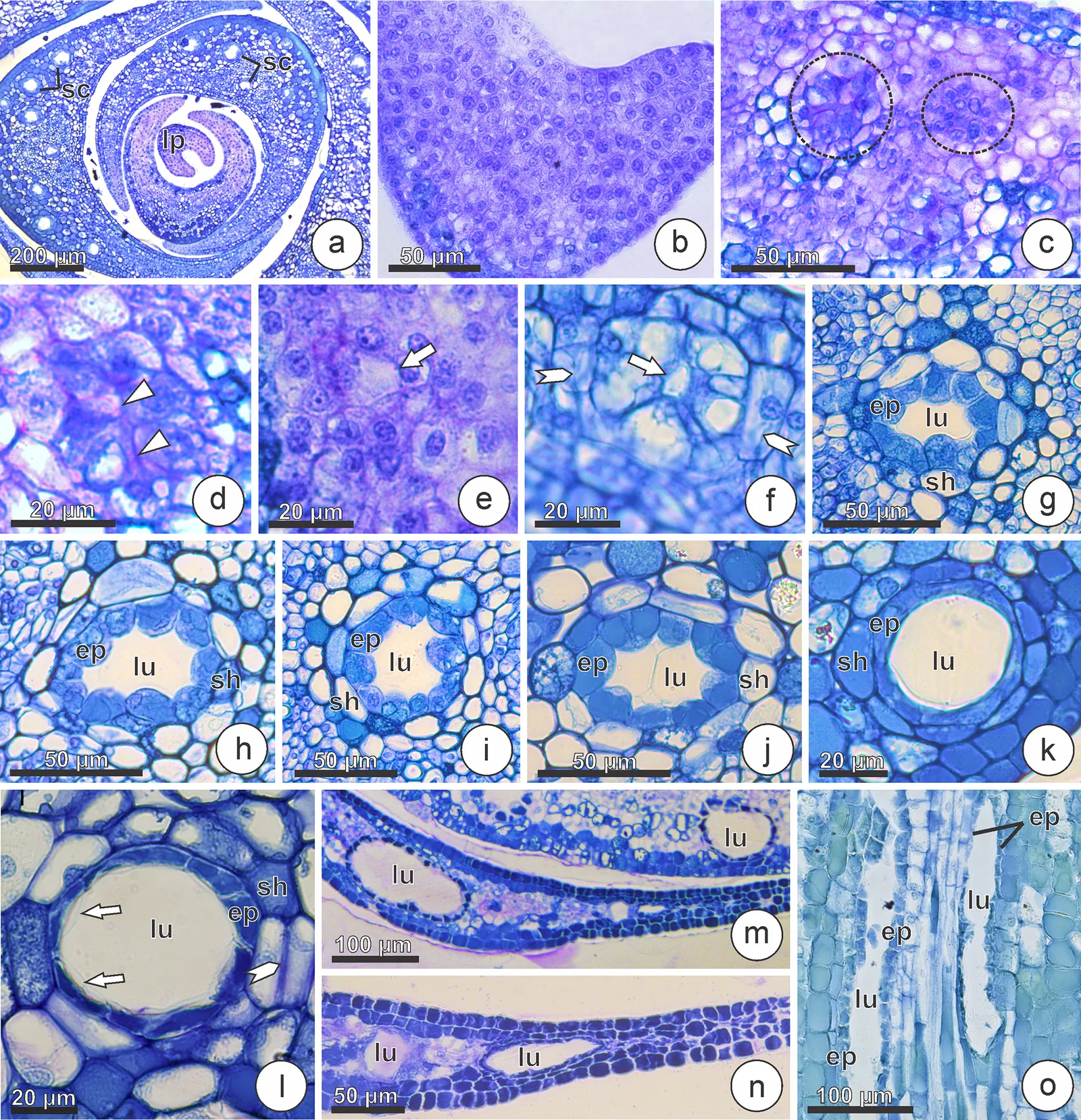
Ontogenesis of secretory cavities of *M. guianensis*. Cross (**a**–**n**) and longitudinal (**o**) sections through the shoot apex protected with cataphylls. (**a**) Compacted, young leaves enclosed by cataphylls. Secretory cavities are present in unexpanded leaves. Central region with leaf primordium. (**b**) Leaf primordia with large cells; conspicuous nuclei and nucleoli. (**c**) Small clusters (circle) of meristematic cells embedded in leaf primordia. (**d**) Clustered pyramidal cells with locally thickened walls (arrowheads). (**e**) Minute triangular intercellular space (arrow) at cluster center. (**f**) Ground meristem with periclinal division (markers), forming 1–2 sheath cell layers. (**g**–**j**) Progressive differentiation of secretory cavity and sheath cells; increased cell volume and vacuolization. (**k**–**l**) Mature stage. (**k**) Mature cavity with spherical lumen; rectangular epithelial cells; sheath cells with dense content. (**l**) Mature cavity with flattened, thin-walled secretory cells showing collapse (arrows); sheath cells with periclinal division. (**m**–**n**) Ovoid and elongated secretory spaces at leaf margin. (**o**) Secretory canals in young stem. *Symbols*: lp = leaf primordium; sc = secretory cavity; ep = epithelial cell; sh = sheath cell; lu = lumen

The dormant apical bud of *M. guianensis* comprises the shoot apical meristem, leaf primordia, and compact, undifferentiated leaves surrounding the apex (Fig. 2a). The initiation and early development of secretory cavities occurred at the initial stages of stem and leaf organogenesis, while these organs remain enclosed within the dormant bud and protected by cataphylls (Fig. 1c). Within these immature organs and tissues, secretory cavities at distinct developmental stages were observed side by side (Fig. 2), revealing an asynchronous pattern of differentiation during early organ development.

Leaf primordia displayed groups of meristematic cells within the ground meristem, characterized by dense cytoplasm and prominent nuclei with conspicuous nucleoli. These cells underwent successive divisions, giving rise to small clusters of tightly packed initial cells (Fig. 2b). Secretory cavities originated from these clusters, which were established early within the ground meristem of the leaf primordia (Fig. 2c). In the central region of these cellular clusters, certain pyramidal cells displayed a conspicuously large nucleus with a prominent nucleolus and locally thickened cell walls (Fig. 2d). These wall thickenings were followed by localized wall degradation, generating a minute intercellular space at the center of the cluster (Fig. 2e). An increase of large cells with conspicuous nuclei was observed (Fig. 2e), preceding progressive protoplast condensation and the release of cellular contents (lysogeny) into the incipient lumen. The initial triangular intercellular space (Fig. 2e) gradually expanded, resulting from localized cell wall separation (schizogeny), characterizing a schizolysigenous mode of cavity formation. During the cavity differentiation, the surrounding epithelial cells underwent radial expansion and progressively delineated the secretory cavity, contributing to the enlargement and structural definition of the lumen (Fig. 2g–j). Simultaneously, cells of the ground meristem adjacent to the developing cavity divided at the periclinal plane (Fig. 2f), giving rise to tangentially elongated or ovoid derivative cells that encircled the cavity (Fig. 2g-k).

The differentiated secretory cavity exhibited circular (Fig. 2k), oval (Fig. 2l, n), or elongated outlines (Fig. 2m) in both transverse and longitudinal sections. Elongated structures were consistently observed in curved or revolute leaf margins (Fig. 2m, n) and young stems (Fig. 2o).

Within the same secretory epithelium, epithelial cells varied in size, shape, and appearance during development. In the undifferentiated cavities, they were larger, with rounded to pyramidal profiles, with a spherical central nucleus and protoplast with a heterogenous aspect, characterized by increase in the cell volume and vacuolization (Fig. 2g–j), whereas in mature cavities they were predominantly flattened and filled with a dense, compact content (Fig. 2k, l). The tangential walls facing the lumen were convex at early developmental stages (Fig. 2g-j) and appeared straight or concave in mature cavities (Fig. 2k, l).

At the mature stage (Fig. 2l), cavities were structurally characterized with a spherical lumen delimited by a uniseriate epithelium with very flat and thin-walled secretory cells with signs of collapse. Ovoid and elongated secretory spaces were observed on the leaf margin (Fig. 2m, n) and young stem (Fig. 2o). The mature cavities were frequently surrounded by a sheath composed of one to two layers of oval to tangentially elongated parenchyma cells (Fig. 2k). The sheath cells displayed dense cytoplasmic contents (Fig. 2k, l), histochemically identified as phenolic compounds. Sheath cells, which were located just below a senescent epithelial cell, often exhibited conspicuous nuclei and underwent periclinal divisions (Fig. 2l).

**Fig. 3:**
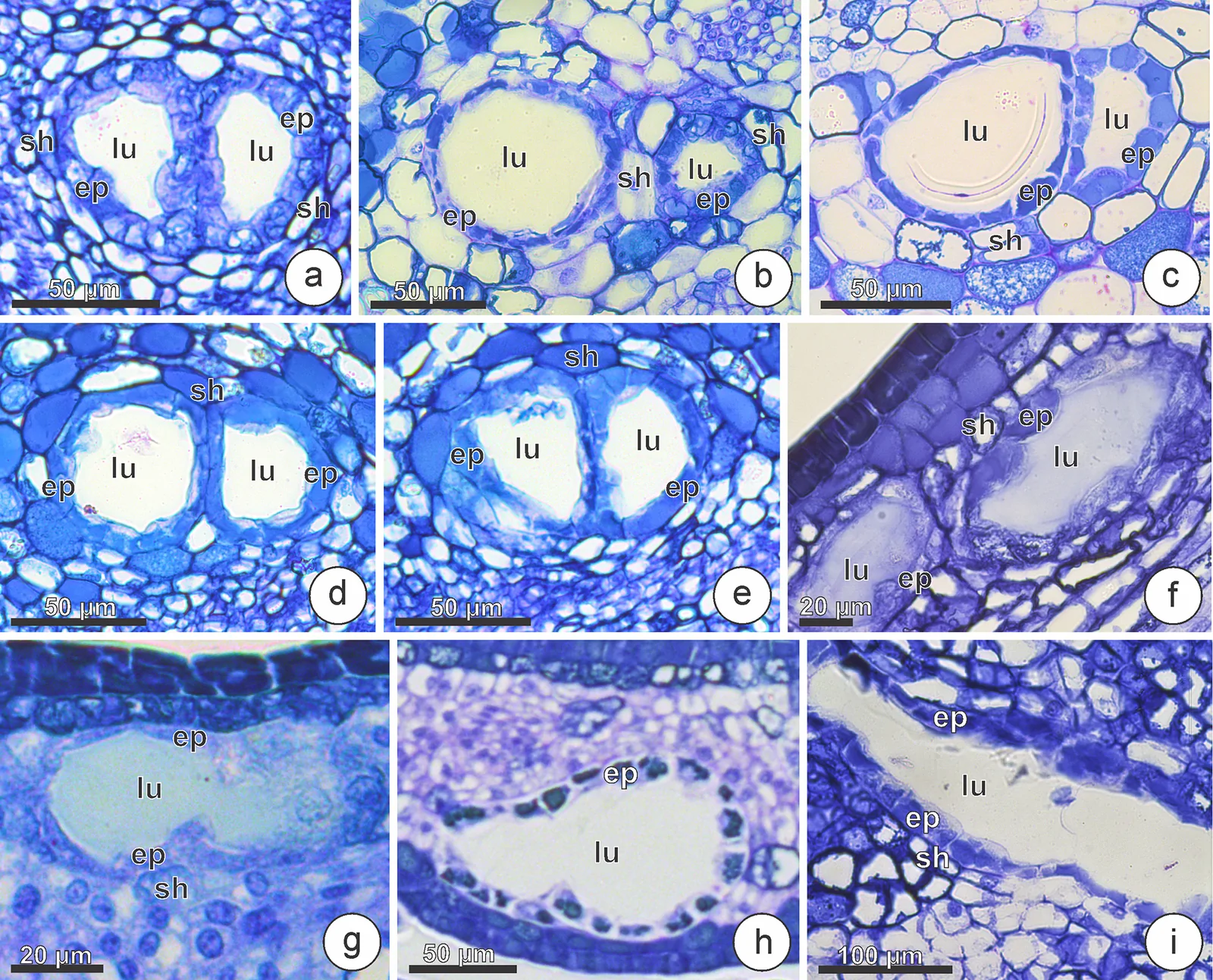
Fusion of secretory cavities of *M. guianensis*. Cross (**a**–**e**) and longitudinal (**f**–**g**, **i**) sections of shoot apex are enclosed by cataphylls. (**a**, **d**–**e**, **h**) Leaf; (**b**, **c**, **f**–**g**, **i**) Stem. (**a**) Juxtaposition of two undifferentiated cavities. (**b**, **c**) Juxtaposition of cavities at distinct developmental stages; in (**b**), note sheath cells separating adjacent cavities. (**d**–**f**) Degeneration of epithelial cells separating adjacent cavities. (**g**–**i**) Single secretory compartment formed by fusion of adjacent cavities; canal-like secretory space; note cell debris within lumen. *Symbols*: ep = epithelial cell; sh = sheath cell; lu = lumen

Juxtaposition was observed between two undifferentiated cavities (Fig. 3a) and between cavities at different developmental stages (Fig. 3b, c). In the initial stages, the juxtaposed undifferentiated cavities remained separated by a wall formed by two layers of epithelial cells (Fig. 3a), each derived from the adjacent cavities, or the cavities were separated by one layer of sheath cells (Fig. 3b). As development progressed, these epithelial layers showed signals of progressive collapse and wall degeneration (Fig. 3d, e), culminating in cavity fusion and the establishment of a single secretory compartment (Fig. 3f-h). Such merged compartments shared a sheath of cells (Fig. 3d, e). In the young portions of the stem, the fusion of adjacent secretory cavities resulted in an extensive and interconnected secretory system (Figs. 2o and 3i). The evidence of ruptured cell walls and cellular debris in the lumen (Fig. 3g-i) suggests recently fused cavities.

**Fig. 4:**
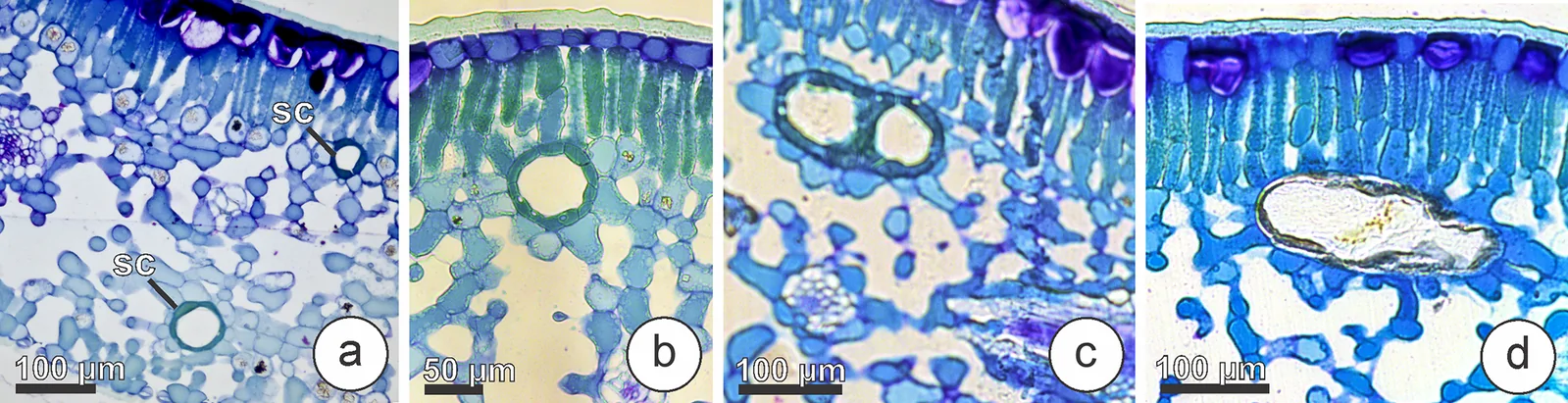
Mature secretory cavities in the expanded leaf of *M. guianensis*. (**a**) Secretory cavities distributed in mesophyll, within palisade and spongy parenchyma. (**b**) Single globose cavity. (**c**) Paired secretory cavities. (**d**) Linear cavity. *Symbol*: sc = secretory cavity

In fully expanded leaves (Fig. 4), mature secretory cavities were embedded within the mesophyll, in both palisade and spongy parenchyma (Fig. 4a). Cavities occurred either isolated (Fig. 4b) or in pairs (Fig. 4c), and exhibited isodiametric (Fig. 4b), ovoid (Fig. 4c) or elongated (Fig. 4d) outlines.

**Fig. 5:**
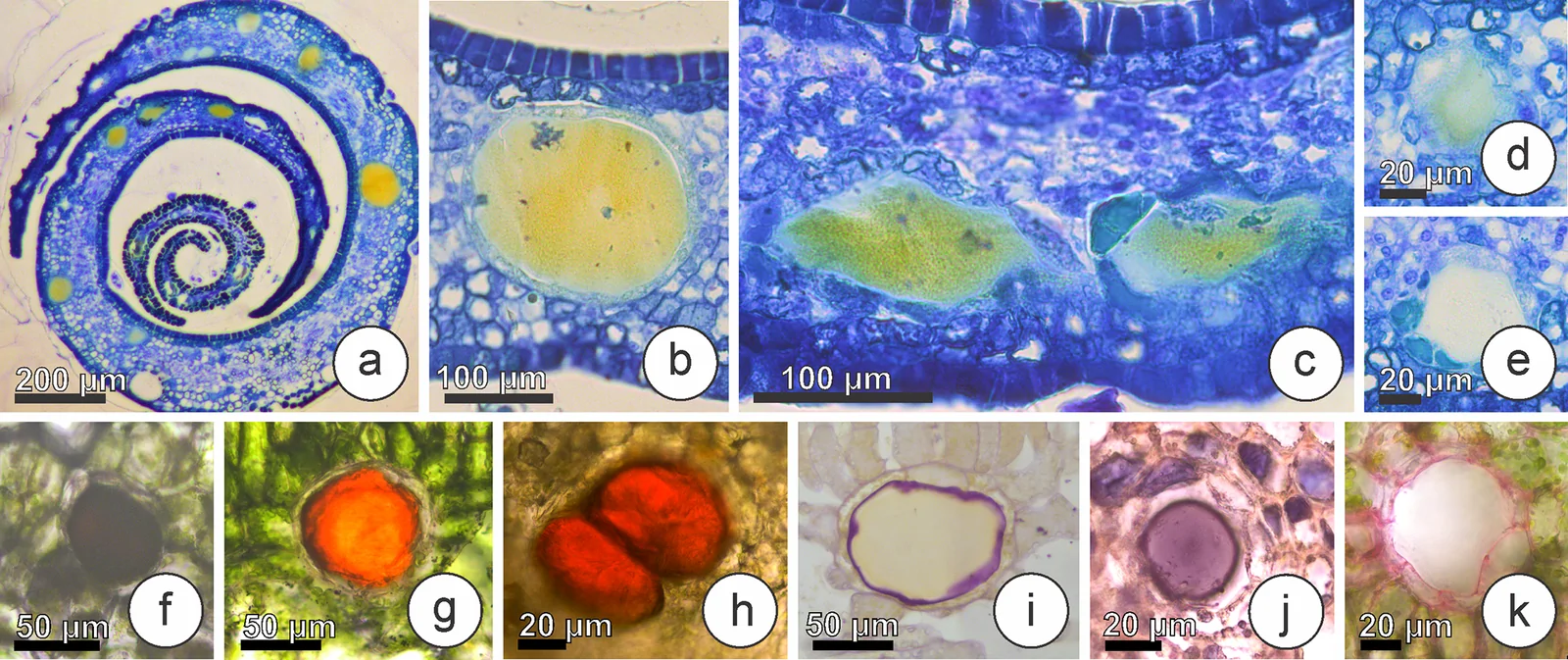
Characterisation of secretions in secretory cavities of *M. guianensis*. Cross sections stained with toluidine blue (**a**–**e**) and histochemical tests (**f**–**k**). (**a**) Cavities containing secretions with distinct aspects and colors. (**b**) Yellowish crystalline luminal content. (**c**–**d**) Yellowish crystalline content associated with opaque, bluish, granular secretion. (**e**) Secretory epithelial cells intensely green with toluidine blue, indicating phenolic compounds. (**f**) Positive ferric chloride reaction; phenolic compounds in lumen. (**g**–**h**) Positive Sudan IV reaction; neutral lipids in lumen of single (**g**) and paired (**h**) cavities. (**i**, **j**) Positive NADI reaction; terpenes (essential oils and oleoresins) as purple deposits at lumen periphery (**i**) or filling lumen (**j**). (**k**) Positive ruthenium red reaction; pectin in epithelial cell walls

Secretory cavities containing abundant secretion were frequently observed in the mesophyll of still undifferentiated leaves and in the young stem stained with toluidine blue (Fig. 5a–d). The lumen content appeared as a yellowish crystalline material (Fig. 5a–d), probably derived from hydroxybenzoquinone. This material was associated with an opaque, bluish, granular secretion, both components occurring concomitantly within the lumen. Some cells of the secretory epithelium, and sheath had a distinct appearance and shape (Fig. 5c, e), and were intensely stained green with toluidine blue, indicating phenolic compounds.

Histochemical tests yielded positive reactions with ferric chloride (Fig. 5f), Sudan IV (Fig. 5g, h), and Nadi reagent (Fig. 5i, j), indicating the presence of phenolic compounds, lipids, and moisture of essential oils and oleoresins, respectively. Epithelial cell walls reacted to ruthenium red treatment (Fig. 5k), indicating the presence of acidic polysaccharides, mainly pectic substances.

## Ultrastructure and secretion dynamics

**Fig. 6:**
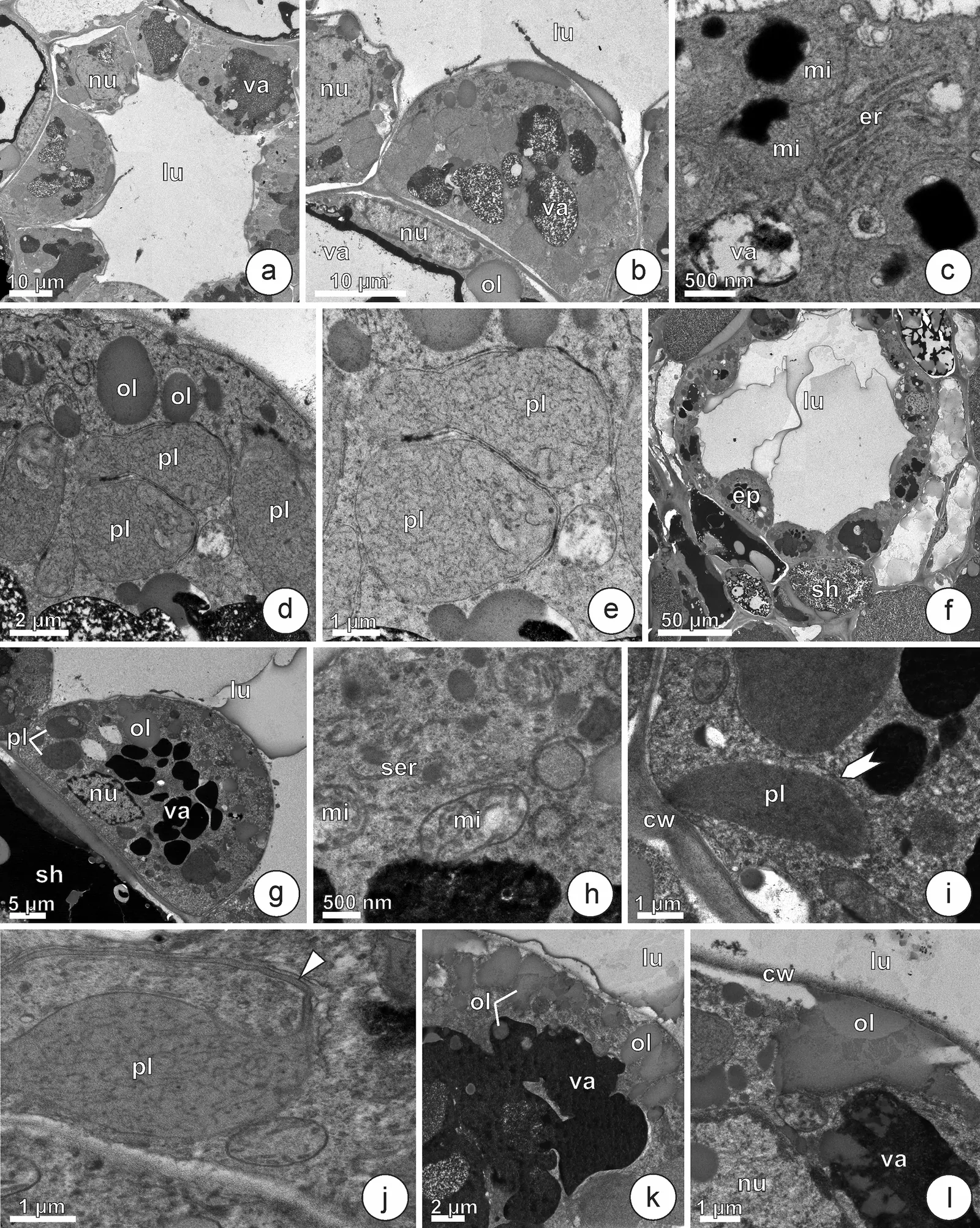
Ultrastructure of the secretory cavities in *M. guianensis*. (**a**–**e**) Differentiating stage; (**f**–**l**) Mature stage. (**a**) Epithelial cells with bulging surface; large nucleus; dense cytoplasm; vacuoles with electron-dense content. (**b**) Numerous small vacuoles with heterogeneous materials; oil bodies at distal pole; nucleus and oil bodies in sheath cell. (**c**) Mitochondria with osmiophilic inclusions; ER tubules with dilated ends; vesicles/prevacuoles with dense content. (**d**) Cytoplasmic oil bodies; plastids with finely granular stroma and membranous network. (**e**) Plastid detail. (**f**) Epithelial cells with variable architecture; secretion in the lumen. (**g**) Bulky cell; peripheral nucleus; small vacuoles with dark inclusions; dense cytoplasm at distal pole. (**h**) Mitochondria and short SER profiles. (**i**) Plastid juxtaposed to plasmalemma; periplastidial reticulum (marker). (**j**) Large plastid with stromule (arrowhead). (**k**) Coalescent vacuoles with dark content; peripheral oil bodies. (**l**) Oil accumulation in periplasmic space. *Symbols*: ep = epithelial cell; sh = sheath cell; lu = lumen; nu = nucleus; va = vacuole; mi = mitochondria; er = endoplasmic reticulum; ol = oil body; pl = plastid; ser = smooth endoplasmic reticulum; cw = cell wall

Under TEM, differentiating secretory cavities exhibited large, pyramidal epithelial cells with a bulging surface facing the lumen (Fig. 6a). These cells were characterised by conspicuous nuclei, a dense and abundant cytoplasm, and a vacuolar system composed of vacuoles differing in shape and number containing electron dense inclusions (Fig. 6b), probably consisting of a mixture of resin and phenolic substances, as indicated by histochemical analyses. At this developmental stage, numerous globular mitochondria with well-developed cristae containing dark inclusions, proliferated endoplasmic reticulum frequently exhibiting dilated tubules, and scattered vesicles with electron-dense contents (Fig. 6c) were commonly observed. In addition, cytoplasmic oil bodies and ovoid plastids filled with finely granular stroma containing a network of thin membranous profiles (Fig. 6d–e) were abundant.

**Fig. 7:**
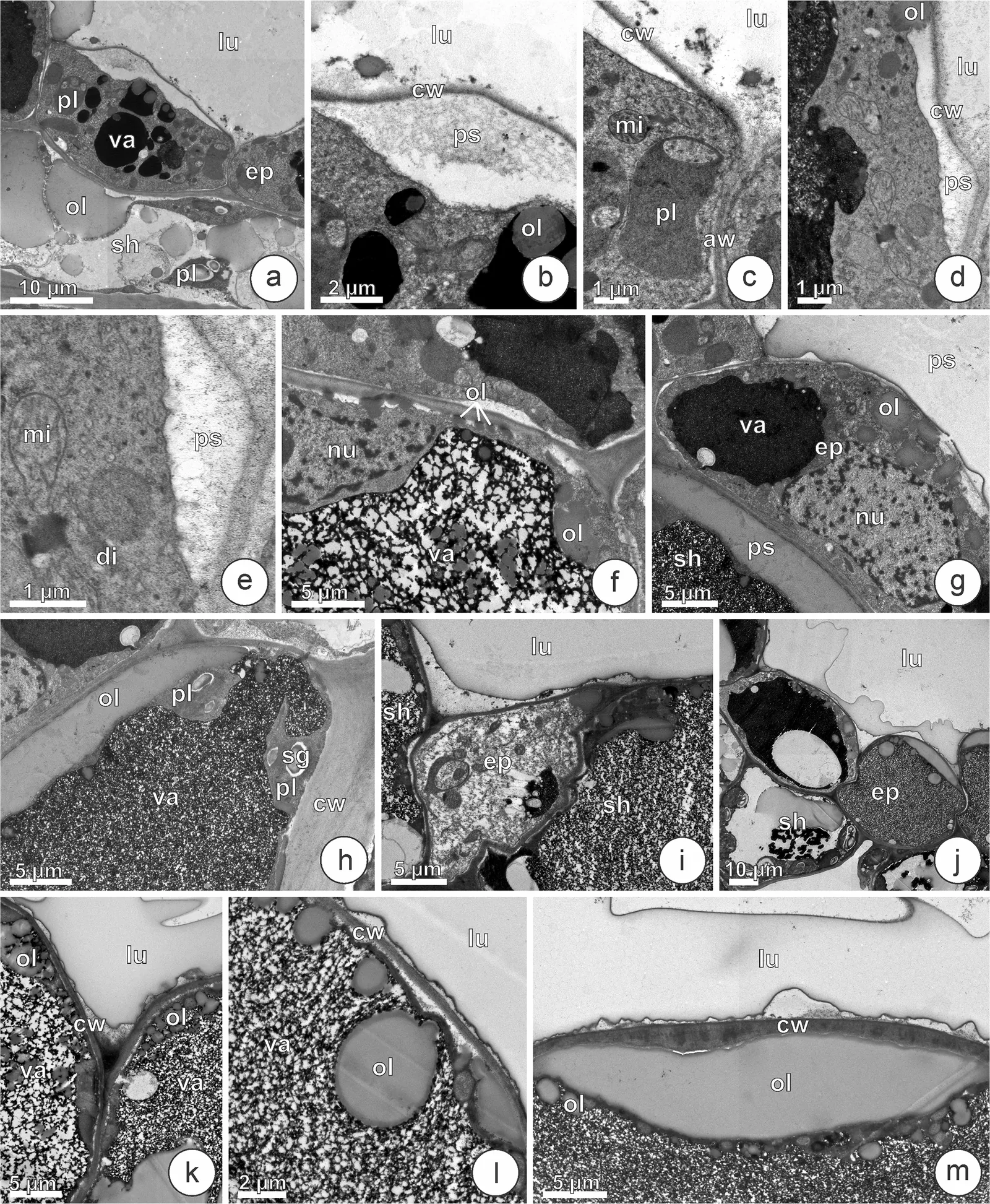
Ultrastructure of the secretory cavities in *M. guianensis*. (**a**) Secretory cell with thin, sinuous, loosened walls; retracted protoplast. (**b**) Enlarged periplasmic space with fibrillar material. (**c**) Thin, sinuous anticlinal walls. (**d**-**e**) Peripheral cytoplasm along anticlinal walls. (**d**) Dense cytoplasm; RER profiles; dictyosome; small oil bodies; vacuole with dense content. (**e**) Polyribosomes; mitochondria; dictyosome; abundant vesicles with electron-dense material; expanded periplasmic space due to protoplast retraction; loosened walls. (**f**) Sheath parenchyma cell; prominent nucleus with heterochromatin; reduced cytoplasm with scattered oil bodies; large central vacuole with electron-dense fibrillar content. (**g**) Sheath cell with oil accumulation in periplasmic space along tangential wall adjacent to epithelium; secretion in lumen. (**h**) Sheath cell with large vacuole containing dark heterogeneous material; chloroplasts with starch grains; thick tangential wall. (**i**) A senescent epithelial cell entrapped by subjacent sheath cells. (**j**) Sheath cells incorporated into secretory epithelium. (**k**) Cellular debris adhered to the cell walls facing the lumen. (**l**) Oil bodies appressed to sheath cell plasmalemma. (**m**) Expanded periplasmic space filled with oil; electron-opaque, predominantly lipidic secretion lining lumen periphery. *Symbols*: ep = epithelial cell; sh = sheath cell; lu = lumen; nu = nucleus; va = vacuole; mi = mitochondria; er = endoplasmic reticulum; ol = oil body; pl = plastid; ser = smooth endoplasmic reticulum; cw = cell wall; ps = periplasmic space; sg = starch grain

As differentiation progressed, the lumen became progressively expanded, and the epithelial cells, varying in size and shape, exhibited ultrastructural heterogeneity. Cells with features indicative of intense metabolic activity (Fig. 6) occurred adjacent to others displaying ultrastructural characteristics consistent with the final stages of the secretory cycle (Fig. 7).

In metabolically active secretory cells, the most conspicuous features included a peripherally positioned, ellipsoidal nucleus and a dense cytoplasm rich in organelles (Fig. 6g). Oil bodies were predominantly concentrated at the distal pole of the cell, whereas small vacuoles contained electron-dense inclusions resembling resin and phenolic material (Fig. 6g). The smooth endoplasmic reticulum (SER) occurred as short, scattered cisternae distributed throughout the cytoplasm (Fig. 6h), while long SER profiles were frequently observed in close association with plastids, forming a periplastidial reticulum (Fig. 6i). Juxtaposition between plastids and plasmalemma was common (Fig. 6i). In some epithelial cells, plastids appeared enlarged and exhibited thin tubular stromal extensions bounded by the envelope membrane (Fig. 6j), corresponding to stromules. Vacuoles displayed irregular and often complex configurations, consistent with fusion events among adjacent compartments (Fig. 6k). These vacuoles contained dark inclusions (resin/phenolic material) and occasional oil bodies. A marked increase in oil bodies was observed in the peripheral cytoplasm at the distal pole of the cells (Fig. 6k), as well as in the periplasmic space of the cell walls facing the lumen (Fig. 6l). Oil droplets were seen traversing the cell wall and ultimately reaching the cavity lumen (Fig. 6l).

Following the secretory process, the epithelial cell walls became sinuous, loosened, and reduced in thickness, while the protoplast underwent retraction (Fig. 7a). A prominent periplasmic space developed along the inner periclinal wall facing the lumen and became filled with fibrillar material (Fig. 7b). The anticlinal walls became thinner, with a loose and lamellar aspect (Fig. 7c). The peripheral cytoplasm was dense and contained polyribosomes, profiles of rough endoplasmic reticulum (RER), mitochondria, small dictyosomes, and abundant vesicles carrying electron-dense material (Fig. 7d, e). Within the expanded periplasmic space formed along the anticlinal walls as a result of protoplast retraction, accumulations of floccular material and osmiophilic granules were observed (Fig. 7e).

At this developmental stage, the sheath cells subjacent to the senescent epithelial cells exhibited a prominent nucleus with heterochromatin, a reduced cytoplasm containing scattered oil bodies, and a large central vacuole filled with electron-dense fibrillar material (Fig. 7f). In other sheath cells, in addition to a large vacuole containing dark fibrillar inclusions, substantial oil accumulations were observed within the periplasmic space along the tangential wall adjacent to the epithelial layer (Fig. 7g). In these cells, plastids bearing starch grains (Fig. 7h) were the distinguishable organelles.

Epithelial cells exhibiting signals of protoplast degeneration appeared to be compressed or entrapped by the subjacent sheath cells (Fig. 7i). The sheath cells were bulky, tangentially elongated or oval, and possessed thicker cell walls. The protoplast was occupied by a prominent vacuole containing electron-dense, heterogeneous material with a granular aspect (presumably phenolic substances histochemically identified) interspersed with oil bodies of varying sizes. These sheath cells were incorporated in the secretory epithelium (Fig. 7j). Cellular debris was observed adhering to the cell walls facing the lumen (Fig. 7k). Oil bodies were accumulated at the distal pole of the cells, closely appressed to the plasmalemma (Fig. 7l), and occurred within an expanded periplasmic space in the same region (Fig. 7m). At this stage, the secretion lining the lumen periphery appeared electron-opaque, homogeneous, and predominantly lipidic.

## Discussion

This study focused on the structural, developmental, and cytological features of the secretory cavities of *M. guianensis*. Although secretory cavities were initially characterized as isolated cavities, the juxtaposition and subsequent fusion of adjacent cavities were common in undifferentiated leaves and stems, demonstrating the occurrence of transitional forms along the organ axis. These intermediate configurations indicated morphological continuity between discrete cavities and elongated structures with a canal-like appearance. Additionally, ultrastructural analyses provide evidence for the integration of sheath cells into the secretory epithelium and their contribution to secretion production.

Although several criteria have traditionally been employed to distinguish secretory canals from cavities, such as the mechanism of lumen formation (lysigenous or schizogenous), overall shape, and longitudinal extension, with canals typically described as elongated intercellular spaces and cavities as more or less spherical or elliptical structures (Evert 2006; Fahn 1979), these features are not always definitive. Our developmental analyses demonstrate that, in *M. guianensis*, the juxtaposition and subsequent anastomosis of adjacent secretory cavities can generate elongated intercellular spaces that closely resemble canal-like structures.

Transitional forms of secretory spaces have been consistently reported (Freitas 2026; Lersten and Curtis 1989), particularly in Leguminosae species (Palermo et al. 2017, 2018), where spherical, ovoid, and elongated cavities may merge or coexist with canals, resulting in a broad spectrum of morphologies within the same organ. A comparable developmental pattern has been described in *Solidago canadensis* (Asteraceae), in which foliar secretory cavities originate as discrete units separated only by epithelial cells (Lersten and Curtis 1989). As development progresses, cavity elongation, together with the stretching and separation of intervening septa, produces the misleading appearance of a single continuous canal rather than a series of interconnected tubular cavities (Lersten and Curtis 1989). This developmental trajectory closely parallels the pattern observed in *M. guianensis*, in which initially discrete cavities undergo progressive juxtaposition and anastomosis, forming elongated intercellular spaces. These findings indicate that canal-like structures may arise secondarily from pre-existing cavities, thereby blurring the classical distinction between canals and cavities based solely on mature morphology. Consequently, our results highlight that a developmental perspective, integrating the analysis of both transverse and longitudinal sections throughout development provides the most reliable basis for the accurate interpretation of secretory cavities and canals.

The occurrence of transitional forms between secretory cavities and canals in *M. guianensis* provides compelling evidence that these structures do not represent discrete categories but rather a morphological continuum shaped during morphogenesis. We interpreted this structural gradation as a plastic response to spatial constraints (e.g., mechanical pressure, space limitation) operating during morphogenesis. The availability of space and physical constraints play a fundamental role in shaping plant cells and organs, and this issue has emerged as an important perspective in developmental botany (Prusinkiewicz and Barbier de Reuille 2010; Ronse De Craene 2018). Experimental evidence indicates that, alongside genetic regulation, modifications in the physical environment of the shoot apical meristem can alter ontogenetic trajectories and, consequently, the morphospace of plant organs (Prusinkiewicz and Barbier de Reuille 2010). Within this framework, the presence of intermediate forms between cavities and canals in *M. guianensis* can be understood as the developmental outcome of spatial displacements and mechanical constraints acting in concert with genetic factors. Together, these complementary perspectives provide a more integrated explanation for the structural diversity and plasticity observed in secretory cavities.

The developmental analyses demonstrated that the secretory cavities derive from fundamental meristem cells and exhibit features characteristic of both lysigenous and schizogenous pathways, supporting their classification as schizolysigenous glands, a pattern reported in different species and even among organs of the same species (Rodrigues and Machado 2009). In agreement with observations on the early onset of secretory activity (Machado et al. 2017), the cavities of *M. guianensis* were active in secretion at the leaf primordium stage, with development extending throughout leaf differentiation.

The ultrastructural organization of the secretory epithelial cells in *M. guianensis* was consistent with that described for cytological processes involved in the synthesis, transport, and compartmentalization of lipophilic secretions (Carvalho et al. 2023, 2025; Cheng et al. 2025; Costa and Demarco 2024; Paiva et al. 2026; Rodrigues and Machado 2009; Sadala-Castilho et al. 2016; Sá-Haiad et al. 2015; Tölke et al. 2021). The plastids features observed in *M. guianensis* appear to play an active role in fatty acid synthesis, generating acyl chains that may be exported and subsequently modified by lipid-synthesising enzymes in the endoplasmic reticulum (Nawrath et al. 2013). The presence of stromules, observed as fine tubular extensions of plastids, suggests intensified interactions between plastids and organelles that may facilitate the exchange of substrates and metabolites required for key biosynthetic pathways (Hanson and Conklin 2020). Similar plastidial projections have been reported in the chloroplasts of extrafloral nectaries, where their close spatial association with the nucleus, mitochondria, endoplasmic reticulum, and plasma membrane supports a role in inter-organelle communication during the secretory process (Machado et al. 2018). Additionally, stromules have been implicated in plant immune responses and nutrient sensing (Gray et al. 2001; Grzyb 2025; Hanson and Conklin 2020). The close association between SER and plastids suggests a highly integrated metabolic network in secretory cells. These coordinated interactions between organelles likely enhance the efficiency of terpene synthesis (such as essential oils and oleorein), transport, and accumulation (Bergman et al. 2024). This underscores the functional specialization of the secretory cavities as active sites for metabolic compartmentalization, intracellular transport, and the production of distinct classes of secondary metabolites. Moreover, the accumulation of lipophilic secretion in the cytoplasm and the periplasmic space of the secretory epithelial cells provides additional evidence of eccrine secretion (Fahn 1979).

During the secretory process, cell wall modifications indicated its active involvement in secretion release. The wall facing the lumen becomes thinner and partially detached, whereas the anticlinal walls remain thicker but exhibit a loose arrangement of cellulose microfibrils. This configuration results in small gaps in which the secretion accumulates prior discharge into the lumen. Cell wall disintegration and the formation of these gaps during resin secretion are closely associated with the activity of lytic enzymes, such as acid phosphatase, involved in wall degradation (Carvalho et al. 2023, 2025). This interpretation is supported by the presence of polyribosomes, RER profiles, dictyosomes, and vesicles in the peripheral cytoplasm along the anticlinal walls of the epithelial cells. Structural wall remodeling, characterized by decreasing wall thickness, microfibril loosening, and periplasmic space formation, together with the pressure generated by secretion accumulation in the periplasmic space, likely facilitates the trans-wall flow of secretion products (Paiva 2016). In addition, the generation of oligosaccharide fragments from degraded wall polysaccharides may enhance plant defense responses against microorganisms (Braga and Dietrich 1987).

Notably, sheath cells adjacent to the degenerating epithelial cells displayed conspicuous nuclei and underwent periclinal divisions, indicating sustained meristematic activity and suggesting that cavity development involves continued peripheral cell recruitment during organ growth. The occurrence of a sheath of cells with meristematic characteristics surrounding secretory canals has been reported in resin glands of several species (Carmello et al. 1995; Lacchia and Carmello-Guerreiro 2009; Machado et al. 2017; Paiva and Machado 2006; Palermo et al. 2017, 2018; Rodrigues et al. 2011a, b; Tölke et al. 2021). TEM analyses demonstrated the replacement of senescent epithelial cells by cells derived from the underlying sheath, accompanied by changes in the ultrastructural appearance of the secretion. The secretion shifted from an electron-dense profile, associated with resin and phenolic compounds, to a predominantly electron-lucent profile, characteristic of oil. This evidence supports the hypothesis that the meristematic sheath plays a key role in the continuous renewal of the secretory epithelium (Rodrigues et al. 2011a), thereby maintaining secretory activity throughout organ development and conferring prolonged functional longevity of these glands.

The coordinated subcellular organization observed in the epithelial cells, particularly the interactions among plastids, endoplasmic reticulum, and mitochondria, as well as cell wall modifications, supports the intense metabolic fluxes required for the synthesis, modification, accumulation, and release of specialized metabolites. Acting synergistically, the identified compounds, mainly terpenes (fundamental components of plant resins) may contribute to the deterrence of herbivores and microbial pathogens, while also enhancing photoprotection by mitigating ultraviolet-induced damage (Abbas et al. 2017; Costa et al. 2020; Harborne 1997; Huo et al. 2013). Terpenoids are among the most diverse and ecologically significant plant secondary metabolites, acting as key defenses against herbivores and pathogens and mediating multiple chemo-ecological interactions. Therefore, the accumulation of diverse classes of substances such as phenolic substances, lipids, oleoresins, and essential oils within secretory structures suggests a conserved metabolic strategy that has been associated with chemical defense (Huo et al. 2013). The synthesis, accumulation, and release of these compounds are tightly associated with specialized secretory structures, whose regulation in response to environmental cues underpins both plant fitness and the biotechnological value of terpenoids (Cheng et al. 2025).

In a broader context, this study provides a structural and developmental basis for understanding the production of bioactive metabolites with ecological, pharmacological and taxonomic significance in Primulaceae.

## Conclusion

This study contributes to the understanding of the diversity of secretory cavities morphotypes side by side that extend beyond the globose and linear secretory structures previously reported (Freitas 2026). These findings challenge the traditional interpretation of these structures as discrete categories and instead support the concept of a developmental continuum shaped by structural plasticity in response to spatial constraints.

In *M. guianensis*, secretory cavities operate as dynamic and integrated structures coordinating organelle activity, cell wall remodeling, and cellular turnover. The coexistence of distinct metabolites within these secretory structures, together with their early differentiation and sustained activity throughout organ maturation, reinforces the interpretation of these structures as multifunctional defensive compartments.

## Data Availability

All data are presented in the article.

## References

[CR1] Abbas F, Ke Y, Yu R, Yue Y, Amanullah S, Jahangir MM, Fan Y (2017) Volatile terpenoids: multiple functions, biosynthesis, modulation and manipulation by genetic engineering. Planta 246:803–816 28803364 10.1007/s00425-017-2749-x

[CR2] Bergman ME, Kortbeek RWJ, Gutensohn M, Dudareva N (2024) Plant terpenoid biosynthetic network and its multiple layers of regulation. Prog Lipid Res 95:101287 38906423 10.1016/j.plipres.2024.101287

[CR3] Braga MR, Dietrich SMC (1987) Defesas químicas de plantas: fitoalexinas. Acta Bot Bras 1:3–16

[CR67] Bueno NR, Castilho RO, Costa RB, Pott A, Pott VJ, Scheidt GN, da Silva Batista M (2005) Medicinal plants used by the Kaiowá and Guaraniindigenous populations in the Caarapó Reserve, Mato Grosso do Sul, Brazil. Acta Bot. Bras. 19: 39-44

[CR4] Carmello SM, Machado SR, Gregorio EA (1995) Ultrastructural aspects of the development in Lithraea molleoides (Vell.) Engl. (Anacardiaceae). Braz J Bot 18:95–103

[CR6] Carvalho SF, Scudeller EL, Santos DC, Machado SR (2023) Intraspecific variation in ultrastructure and secretion of the resin canals in *Anacardium humile* (Anacardiaceae). Protoplasma 260:919–934 36447072 10.1007/s00709-022-01823-5

[CR5] Carvalho SF, Scudeler EL, Machado SR (2025) Microscopy-based methods for characterizing autophagy and understanding its dynamics in resin secretion. Micron 192–193:103818 40138762 10.1016/j.micron.2025.103818

[CR7] Cheng R, Yang S, Wang D, Qin F, Wang S, Meng S (2025) Advances in the biosynthesis of plant terpenoids: models, mechanisms, and applications. Plants 14:1428 40430993 10.3390/plants14101428 PMC12114759

[CR8] Costa ER, Demarco D (2024) Development and holocrine secretion of resin ducts in *Kielmeyera appariciana* (Calophyllaceae). Plants 13:1757 38999597 10.3390/plants13131757 PMC11243538

[CR9] Costa LFPB, Contini SHT, Teixeira SP, Freitas MF, França SC, Bertoni BV, Pereira AMS (2020) Secretory structures and chemical composition of the essential oil from leaves of Myrsine leuconeura Mart. (Primulaceae). Flora 262:151496

[CR69] Ronse De Craene L (2018) Understanding the role of floral development in the evolution of angiosperm flowers: clarifications from a historical and physico-dynamic perspective. J Plant Res131:367-393 10.1007/s10265-018-1021-1 29589194

[CR10] David R, Carde JP (1964) Histochimie-coloration differentielle des inclusions lipidiques et terpeniques des pseudophylles du pin maritime au moyen du reactif NADI. CR Hebd Seances Acad Sci 258:1338

[CR11] De Luna BN, Defaveri ACAE, Sato A, Bizzo HR, Freitas MF, Barros CF (2014) Leaf secretory tissues in *Myrsine coriacea* and *Myrsine venosa* (Primulaceae): ontogeny, morphology, and chemical composition of essential oils. Botany 92:757–766

[CR13] De Luna BN, Freitas MF, Baas P, De Toni KLG, Barros CF (2017) Leaf anatomy of five Neotropical genera of Primulaceae. Int J Plant Sci 178:362–377

[CR12] De Luna BN, Freitas MF, Barros CF (2019) Diversity of leaf secretory structures in five Neotropical genera of Primulaceae: ecological aspects and evolutionary significance. Botany 97:35–51

[CR60] De Ronse L (2018) Understanding the role of floral development in the evolution of angiosperm flowers: clarifications from a historical and physico-dynamic perspective. J Plant Res 131:367–393 29589194 10.1007/s10265-018-1021-1

[CR14] Evert RF (2006) Esau’s plant anatomy; meristems, cells and tissues of the plant body their structure, function and development. Wiley, New York

[CR15] Fahn A (1979) Secretory tissues in plants. Academic, London

[CR16] Fahn A (1988) Secretory tissues in vascular plants. New Phytol 108:229–257 33873942 10.1111/j.1469-8137.1988.tb04159.x

[CR17] França H, Corrêa AL, Oliveira AP, Kuster RM, Santos RP, Rocha L (2011) Flavonoids from *Myrsine rubra* M. F. Freitas & Kinoshita (Myrsinaceae). Biochem Syst Ecol 39:885–887

[CR18] Franco JR, Dal Pai E, Calça MVC, Raniero MR, Dal Pai A, Sarnighausen VCR, Sanchéz-Román RM (2023) Atualização da normal climatológica e classificação climática de Köppen para o município de Botucatu-SP. Irriga 28:77–92

[CR68] Freitas MF (2012) Primulaceae. In: Vibrans, A.C.; Sevegnani, L.; Gasper, A.L. & Lingner, D.V. (eds.) Inventário Florístico Florestal de Santa Catarina. Vol. 1. Editora da FURB, Blumenau. pp 307-309

[CR21] Freitas MF (2026) *Myrsine* in Flora e Funga do Brasil. Jardim Botânico do Rio de Janeiro. https://floradobrasil.jbrj.gov.br/FB10223. Access 02 Feb. 2026

[CR19] Freitas MF, Carrijo TT (2008) A família Myrsinaceae nos contrafortes do Maciço da Tijuca e entorno do Jardim Botânico do Rio de Janeiro. Brasil Rodriguésia 59:813–828

[CR20] Freitas MF, Kinoshita LS (2015) *Myrsine* (Myrsinoideae-Primulaceae) no sudeste e sul do Brasil. Rodriguésia 66:167–189

[CR22] Gottsberger G, Silberbauer-Gottsberger I (2006) Life in the Cerrado: a South American Tropical seasonal ecosystem. Vol. I. Origin, Structure, Dynamics and Plant Use. Reta, Ulm

[CR23] Gray J, Sullivan J, Hibberd J, Hansen M (2001) Stromules: mobile protrusions and interconnections between plastids. Plant Biol 3:223–233

[CR24] Grzyb J (2025) Stromules: an incident or formalized pathway for molecules transfer between organelles? Int J Mol Sci 26:10680 41226714 10.3390/ijms262110680 PMC12608775

[CR25] Hanson MR, Conklin PL (2020) Stromules, functional extensions of plastids within the plant cell. Curr Opin Plant Biol 58:25–32 33137706 10.1016/j.pbi.2020.10.005

[CR26] Harborne JB (1997) Biochemical plant ecology. In: Dey PM, Harborne JB (eds) Plant biochemistry. Academic, London, pp 503–516

[CR27] Huot OB, Nachappa P, Tamborindeguy C (2013) The evolutionary strategies of plant defenses have a dynamic impact on the adaptations and interactions of vectors and pathogens. Insect Sci 20:297–306 23955882 10.1111/1744-7917.12010

[CR28] Jensen WA (1962) Botanical histochemistry: principles and practice. W. H. Freeman and Company, San Francisco

[CR29] Johansen DA (1940) Plant microtechnique. McGraw-Hill, New York

[CR30] Judd WS, Campbell CS, Kellogg EA, Stevens PF, Donoghue MJ (2009) Plant systematics: a phylogenetic approach. Sinauer Associates Inc., Sunderland

[CR31] Karnovsky MJ (1965) A formaldehyde-glutaraldehyde fixative of high osmolality for use in electron microscopy. J Cell Biol 27:137–138

[CR32] Lacchia APS, Carmello-Guerreiro SM (2009) Aspectos ultra-estruturais dos canais secretores em órgãos vegetativos e reprodutivos de Anacardiaceae. Acta Bot Bras 23:376–388

[CR33] Lersten NR, Curtis JD (1989) Foliar oil reservoir anatomy and distribution in Solidago canadensis (Asteraceae, tribe Astereae). Nord J Bot 9:281–287

[CR36] Machado SR, Carmello-Guerreiro SM (2001) Estrutura e desenvolvimento de canais secretores em frutos de *Schinus terebinthifolius* Raddi (Anacardiaceae). Acta Bot Bras 15:189–195

[CR35] Machado SR, Canaveze Y, Rodrigues TM (2017) Structure and functioning of oil cavities in the shoot apex of *Metrodorea nigra* A. St -Hil (Rutaceae) Protoplasma 254:1661–1674 27957603 10.1007/s00709-016-1056-x

[CR37] Machado SR, Gregório EA, Rodrigues TM (2018) Structural associations between organelle membranes in nectary parenchyma cells. Planta 247:1067–1107 29344723 10.1007/s00425-018-2844-7

[CR34] Machado SR, Bento KBD, Canaveze Y, Rodrigues TM (2023) Peltate trichomes in the dormant shoot apex of *Metrodorea nigra*, a Rutaceae species with rhythmic growth. Plant Biol 25:161–175 36278887 10.1111/plb.13480

[CR38] Martínez–Quezada DM, Rojas–Leal A, Villaseñor JL, Terrazas T (2025) Structural considerations and differences between leaf canals and secretory cavities in Asteraceae. Protoplasma 262:707–720 39805992 10.1007/s00709-024-02028-8 PMC12018624

[CR39] Metcalfe CR, Chalk L (1950) Anatomy of the Dicotyledons: leaves, stem and wood in relation to taxonomy with notes on economic uses. Vols. I, II. Clarendon, Oxford

[CR40] Moges GW, Manahelohe GM, Assege M, Tasew BS, Molla DK, Belew AA (2025) Phytochemical profiles and biological activity of *Myrsine africana* L.: a comprehensive review. Front Pharmacol 16 10.3389/fphar.2025.1565656 PMC11965677 40183106

[CR41] Nawrath C, Schreiber L, Franke RB, Geldner N, Reina-Pinto JJ, Kunst L (2013) Apoplastic diffusion barriers in *Arabidopsis*. Arabidopsis Book 11:e0167 24465172 10.1199/tab.0167 PMC3894908

[CR42] O’Brien TP, Feder N, McCully ME (1964) Polychromatic staining of plant cell walls by toluidine blue. Protoplasma 59:368–373

[CR43] Ogawa H, Natori S (1968) Hidroxybenzoquinones from Myrsinaceae plants–II.–Phytochemistry 7:773–785

[CR45] Otegui M, Maldonado S (1998) Morfologia foliar de las especies de *Myrsine* L. (Myrsinaceae) del Cono Sur de America del Sur. Candollea 53:349–363

[CR44] Otegui M, Lima C, Maldonado S, Lederkremer RM (1998a) Histological and chemical characterization of *Myrsine laetevirens* Seed. Int J Plant Sci 159:762–772

[CR46] Otegui MS, Gaspar ML, Maldonado S, Varetti EL, Pollero R (1998b) Studies on tissues associated to hydroxybenzoquinone secretion in *Myrsine laetevirens* (Myrsinaceae). Nord J Bot 18:447–459

[CR49] Paiva EAS (2016) How do secretory products cross the plant cell wall to be released? A new hypothesis involving cyclic mechanical actions of the protoplast. Ann Bot 117:533–540 26929201 10.1093/aob/mcw012 PMC4817504

[CR47] Paiva EAS, Machado SR (2006) Structural and ultrastructural aspects of ontogenesis and differentiation of resin secretory cavities in *Hymenaea stigonocarpa* (Fabaceae, Papilionoideae) leaves. Nord J Bot 24:423–431

[CR48] Paiva EAS, Oliveira DMT, Leite VG, Teixeira SP (2026) Morphology and secretory activity of resin-secreting glands in the fruits of *Myroxylon peruiferum* (Leguminosae, Papilionoideae). Flora 336:152910

[CR51] Palermo FH, Teixeira SP, Mansano VF, Leite VG, Rodrigues TM (2017) Secretory spaces in species of the clade *Dipterygeae* (Leguminosae, Papilionoideae). Acta Bot Bras 31:374–381

[CR50] Palermo FH, Rodrigues MIA, Nicolai J, Machado SR, Rodrigues TM (2018) Resin secretory canals in *Protium heptaphyllum* (Aubl.) Marchand. (Burseraceae): a tridimensional branched and anastomosed system. Protoplasma 255:899–910 29264702 10.1007/s00709-017-1197-6

[CR52] Pizzolato P, Lillie RD (1973) Mayer’s tannic acid-ferric chloride stain for mucins. J Histochem Cytochem 21:56–64 4121044 10.1177/21.1.56

[CR53] Prado E, Demarco D (2018) Laticifers and secretory ducts: similarities and differences. In: Hufnagel L (ed) Ecosystem services and global ecology. InTech, London, pp 103–123

[CR54] Prusinkiewicz P, Barbier de Reuille P (2010) Constraints of space in plant development. J Exp Bot 61:2117–2129 20388746 10.1093/jxb/erq081

[CR55] Reynolds ES (1963) The use of lead citrate at high pH as an electron opaque stain in electron microscopy. J Cell Biol 17:208–212 13986422 10.1083/jcb.17.1.208 PMC2106263

[CR56] Rodrigues TM, Machado SR (2009) Developmental and structural features of secretory canals in root and shoot wood of *Copaifera langsdorffii* Desf. (Leguminosae, Caesalpinioideae). Trees 23:1013–1018

[CR57] Rodrigues TM, Machado SR (2012) Oil glands in *Pterodon pubescens* Benth (Leguminosae–Papilionoideae): distribution, structure and secretion mechanisms. Int J Plant Sci 173:984–992

[CR58] Rodrigues TM, Santos DC, Machado SR (2011a) The role of the parenchyma sheath and PCD during the development of oil cavities in *Pterodon pubescens* (Leguminosae-Papilionoideae). C R Biol 334:535–543 21784363 10.1016/j.crvi.2011.04.005

[CR59] Rodrigues TM, Teixeira SP, Machado SR (2011b) The oleoresin secretory system in seedlings and adult plants of copaíba (*Copaifera langsdorffii* Desf., Leguminosae–Caesalpinioideae). Flora 206:585–594

[CR62] Sá-Haiad B, Silva CP, Paula RCV, Rocha JF, Machado SR (2015) Androecia in two *Clusia* species: development, structure and resin secretion. Plant Biol 17:816–882 25678221 10.1111/plb.12314

[CR61] Sadala-Castilho R, Machado SR, Sá-Haiad B, Lima HA (2016) Oil-resin glands in Velloziaceae flowers: structure, ontogenesis and secretion. Plant Syst Evol 302:585–599

[CR63] Thoa NT, Son NT (2024) The genus *Myrsine*: a review of phytochemistry, pharmacology, and toxicology. Fitoterapia 177:106121 38992476 10.1016/j.fitote.2024.106121

[CR64] Tölke ED, Lacchia APS, Lima EA, Demarco D, Ascensao L, Carmello- Guerreiro SM (2021) Secretory ducts in Anacardiaceae revisited: updated concepts and new findings based on histochemical evidence. S Afr J Bot 138:394–405

[CR65] Turner GW, Berry AM, Gifford EM (1998) Schizogenous secretory cavities of *Citrus limon* (L.) Burm. F. and a reevaluation of the lysigenous gland concept. Int J Plant Sci 159:75–88

[CR66] Watson ML (1958) Staining of tissue sections for electron microscopy with heavy metals. J Biophys Biochem Cytol 4:475–478 13563554 10.1083/jcb.4.4.475 PMC2224499

